# What is the promise of personalised nutrition?

**DOI:** 10.1017/jns.2021.13

**Published:** 2021-04-06

**Authors:** Paola G. Ferrario, Bernhard Watzl, Grith Møller, Christian Ritz

**Affiliations:** 1 Department of Physiology and Biochemistry of Nutrition, Max Rubner-Institut, Karlsruhe, Germany; 2 Department of Nutrition, Exercise and Sports, University of Copenhagen, Frederiksberg, Denmark

**Keywords:** Classification, Outcome-based, Population reference, Regression models, Statistical learning

## Abstract

Personalised nutrition (PN) is an emerging field that bears great promise. Several definitions of PN have been proposed and different modelling approaches have been used to claim PN effects. We tentatively propose to group these approaches into two categories, which we term outcome-based and population reference approaches, respectively. Understanding the fundamental differences between these two types of modelling approaches may allow a more realistic appreciation of what to expect from PN interventions presently and may be helpful for designing and planning future studies investigating PN interventions.

## Introduction

Presently, there is much hype about personalised nutrition (PN), both in nutritional research and in the food and nutrients industry. In the former, it comes with a promise to change how nutrition-related health problems may be addressed, possibly prevented, or even solved through targeted nutritional interventions. In the latter, it comes with a promise of big business perspectives. But what does PN actually mean and what to expect from it? Therefore, it is not surprising that studies discussing how to define PN benefits and effects are being published all the time^(1–8)^.

A crucial point that seems not to have received much attention though, is the data science and/or statistical methodological framework used for leveraging PN effects from individual data. The aim of this brief report is to tentatively suggest a taxonomy for characterising the data analytic approaches used for claiming PN effects. Such a characterisation may help better appreciate what presently can and cannot be realistically expected from PN.

## Methods

In the following paragraphs, we describe and exemplify two types of data analytic approaches that may be useful for characterising studies that claim PN effects. We have termed these two types of approaches, outcome-based and population reference, respectively, reflecting how they utilise individual data.

### Outcome-based approaches

**Fig. 1.**
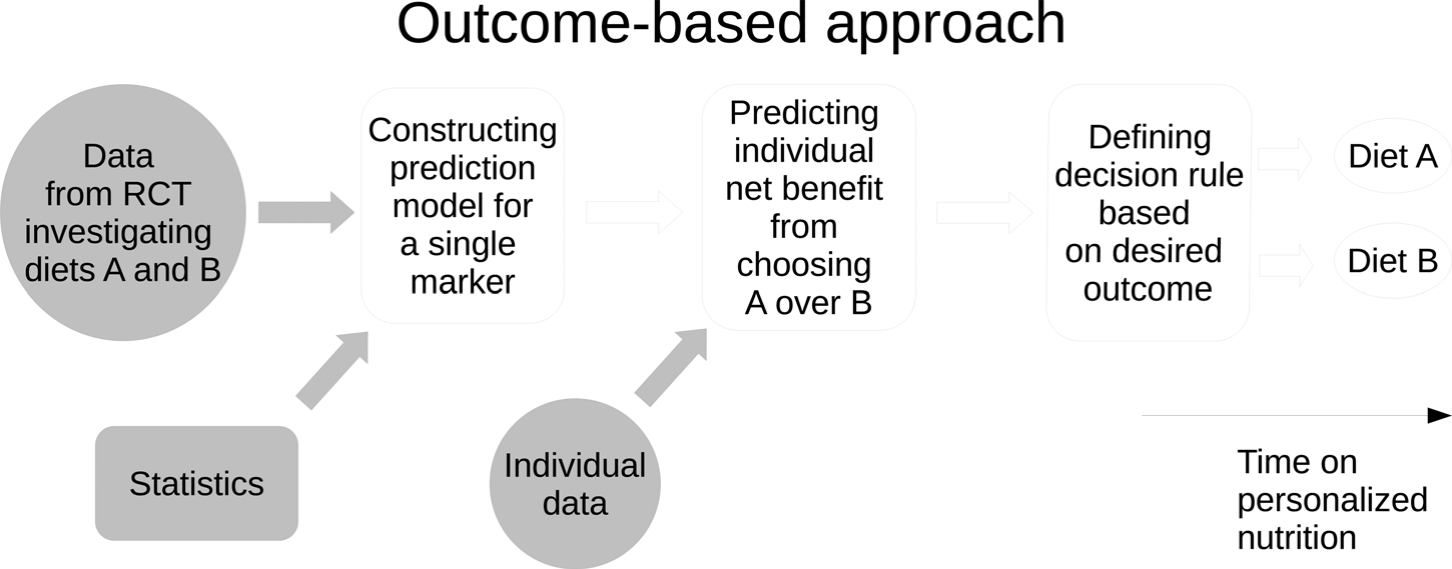
Conceptual diagram showing the steps in the outcome-based approach.

The outcome-based approach targets a concrete, pre-specified quantitative improvement in a specific health outcome such as weight loss or improvement in micronutrient status. Specifically, the PN effect is defined as the change in the health outcome achieved by choosing one well-defined diet (for a certain time period) over another well-defined (reference) diet in such a way that the change for any individual is determined by certain biomarker levels of the individual, which are available at a certain time point. This means that the PN effect will be a function of the biomarker(s) considered. Conceptually, outcome-based approaches resemble precision medicine where several biomarker-driven decision rules for choosing one treatment over another treatment have been proposed^(9–11)^. A key feature is that outcome-based approaches are not adaptive to changes in individual data: Individual biomarker data, such as baseline data, are provided upfront, and then, it is decided which diet to follow for a certain time period. The key concepts and inputs of the population reference approach are shown in Figure 1.

To be able to predict such PN effects, data are needed from comparative studies where both the health outcome and the biomarker(s) were measured while comparing the diets. Such studies will typically be randomised controlled trials (RCTs) where biomarkers were measured as part of a baseline assessment. Utilising such existing data, suitable prediction models may be fitted and, subsequently, used for the prediction of PN effects for any individuals for which the relevant biomarker data are available. Specifically, the resulting prediction models take individual biomarker data as input and provide the expected individual change in the health outcome as output. Naturally, we will be most interested in PN effects that lead to improvements in health outcomes. However, for some biomarker data configurations, the PN effect may indeed be a negligible improvement or, possibly, even detrimental.

### Population reference approaches

**Fig. 2.**
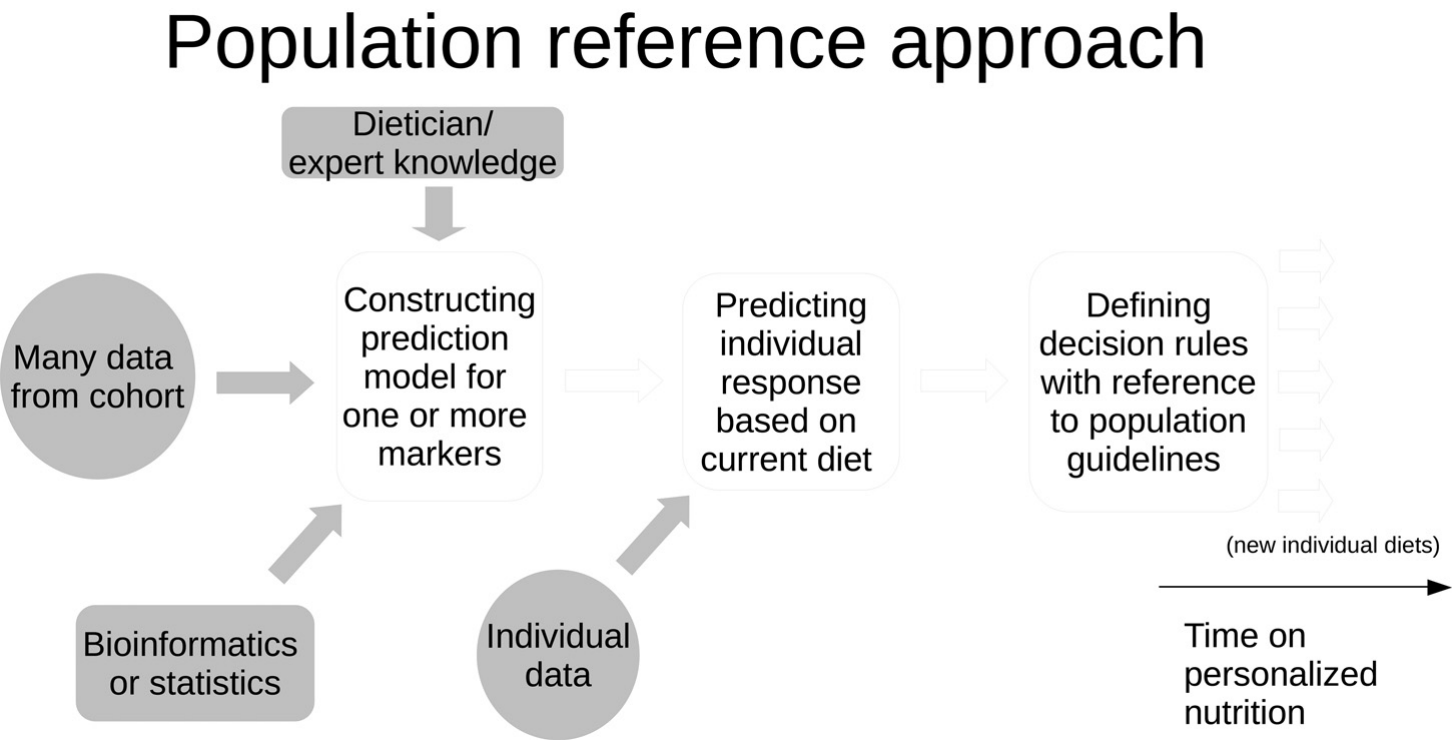
Conceptual diagram showing the steps in the population reference approach.

The population reference approach seeks to establish an overall improvement in the health status for several health outcomes. For each individual, this is achieved through an evaluation of individual biomarker and other data and acting upon any departures from well-defined population-level dietary guidelines and recommendations through the provision of dietary advice, suggesting changes towards a more healthy diet. Naturally, different individuals may receive different (PN) pieces of dietary advice. Also, the more data are used the more likely it is that different individuals will receive different pieces of advice. The key concepts and inputs of the population reference approach are shown in Figure 2.

The evaluation of data may be carried out (1) manually based on expert knowledge provided by dieticians or nutritionists and/or (2) by means of bioinformatics and/or statistical methods. In both cases, the evaluation may be guided by a number of decision rules on which advice to provide under which circumstances. The former limits the amount of individual data that may be processed, whereas the latter involves some kind of data reduction and, consequently, it may utilise the large amounts of individual data. The PN effect resulting from following a diet that has been guided by multiple pieces of PN advice over a certain time period is defined as the improvement in the general health status as measured through one or more outcomes. In case new data become available at a later time point, they may be utilised for updating any advice already given.

To be able to establish proof of concept of PN effects obtained from population reference approaches, RCTs may be carried out to provide data for comparing an intervention based on PN advice with a standard dietary intervention or standard of care intervention.

## Results and discussion

### Outcome-based approaches

Two recent studies have explored PN effects in terms of increased weight loss from following a so-called New Nordic Diet instead of following a standard Western diet for half a year based on either a single phenotypic (fasting plasma glucose) or a single genetic biomarker (the ratio between Prevotella and Bacteroides gut bacteria)^(12,13)^. Specifically, the increased weight loss obtained from following the New Nordic diet compared with following the standard Western diet for 26 weeks was shown, using a linear mixed model with a biomarker-diet interaction, to depend linearly on the baseline fasting plasma glucose level: the higher the glucose level at the baseline, the larger the additional weight loss due to the New Nordic diet after 26 weeks^(12)^.

In principle, the outcome-based approach might utilise many different types of data simultaneously and not just a single biomarker. However, if the approach relies on structure imposed through regression modelling as was the case in the two above examples, then using more data becomes much more challenging and indeed a topic of ongoing research in the statistical literature^(11)^. Moving beyond regression models towards grey- and black box modelling using other data analytic tools such as machine learning and random forests would be an interesting alternative. Sticking to fairly simple regression models also has its charm as it would allow easy comparison between studies or even enable combining results across studies and it would also help ensure reproducibility, two aspects that may be useful at present in advancing PN.

Outcome-based approaches rely heavily on empirical or mechanistic models that have already established the link between biomarker information and the outcome of interest. Data from RCTs, which have already been conducted, may be revisited and re-analysed (in various ways) to provide such models. Thus, in a first step, it does not require running new trials as already existing data can be exploited, potentially providing novel insights at a low cost. Moreover, whether or not the RCT resulted in a positive result in the first place is not important, as there could be biomarker-specific effects in any case^(10)^. RCTs could also be planned to investigate PN effects directly, but to our knowledge, no such RCT has yet been conducted.

In analogy to personalised medicine, outcome-based approaches could also be extended to provide binary decision rules for when to recommend one diet over another diet for achieving a certain improvement.

### Population reference approaches

Recently, two studies applied population reference approaches for predicting glycaemic response and plasma glucose, respectively, based on individual data including food intake^(14,15)^. Specifically, individual glycaemic and postprandial responses were predicted from individual data, such as baseline characteristics, single-nucleotide polymorphisms, gut-microbiome features and habitual diet, by means of multivariate linear regression and a machine learning method (boosting), respectively. Predicted responses were correlated against observed responses, but no decision rules for new altered diets were derived.

However, in several other recent studies, decision rules were applied: Individuals would receive expert- and/or machine learning-based personal counselling as compared with standard ‘one fits all’ advice in order to change dietary habits^(16–18)^. For instance, if fat intake was (predicted to be) higher than recommended, the personalised counselling suggested changes in the diet aiming to reduce fat intake, such as ‘Limit your intake of saturated fats found in butter, full-fat dairy products and processed foods e.g. biscuits, pastries and processed meats’^(16)^.

There are very many options for deriving data-driven decision rules as the ones used in some of the above-mentioned articles. In principle, it may be any method capable of classification, including statistical regression models, random forests and machine learning methods. There is no easy way subsequently to unravel the separate effects (on any specific outcome) resulting from these pieces of PN dietary advice. For each individual, it would require very detailed and meticulous bookkeeping of all pieces of advice provided during the time period in question, including time stamps, duration and applied decision rule(s). Subsequently, it would also require a diligent use of statistical methods to quantify the effects of pieces of personalised dietary advice. The more individual data were used for providing the PN dietary advice, the more difficult it will be to unravel the separate effects. Consequently, repeating an RCT involving a population reference approach will not necessarily provide comparable results as different individuals will most likely receive different sequences of PN dietary advice.

### Perspective

In PN, population reference approaches seem to be preferred over outcome-based approaches. One reason may be that population reference approaches seem capable of utilising vast amounts of data but are presently not so principled, and the generalisation of results is difficult. There is, however, a need to better characterise and understand the PN effects that are presently reported, as a way of improving reproducibility and generalizability. On the other hand, outcome-based approaches utilise much less data but are more principled and provide results that seem to generalise more readily. This simplicity comes at the price of restrictive modelling assumptions and the limited use of individual data. Therefore, there is a need for more flexible extensions.

Finally, presently, the implementations of both types of approaches ignore that PN is about benefits or effects at an *individual level*, whereas applied data analytic methods were developed for estimating and evaluating *population-level* effects. Consequently, these methods cannot be used alone if results should be applicable at an individual level rather than a population level. Instead, they need to be augmented or modified to become useful. This means that the classical statistical inference paradigm must be replaced by a much more recent statistical learning framework, which, among other things, involves the use of concepts such as prediction models and cross-validation^(19,20)^.
